# Understanding Immersivity: Image Generation and Transformation Processes in 3D Immersive Environments

**DOI:** 10.3389/fpsyg.2012.00284

**Published:** 2012-08-10

**Authors:** Maria Kozhevnikov, Rupali P. Dhond

**Affiliations:** ^1^Psychology Department, National University of SingaporeSingapore; ^2^Department of Radiology, Martinos Center for Biomedical Imaging, Harvard Medical SchoolCharlestown, MA, USA

**Keywords:** mental rotation, immersivity, three-dimensional immersive virtual environments

## Abstract

Most research on three-dimensional (3D) visual-spatial processing has been conducted using traditional non-immersive 2D displays. Here we investigated how individuals generate and transform mental images within 3D immersive (3DI) virtual environments, in which the viewers perceive themselves as being surrounded by a 3D world. In Experiment 1, we compared participants’ performance on the Shepard and Metzler (1971) mental rotation (MR) task across the following three types of visual presentation environments; traditional 2D non-immersive (2DNI), 3D non-immersive (3DNI – anaglyphic glasses), and 3DI (head mounted display with position and head orientation tracking). In Experiment 2, we examined how the use of different backgrounds affected MR processes within the 3DI environment. In Experiment 3, we compared electroencephalogram data recorded while participants were mentally rotating visual-spatial images presented in 3DI vs. 2DNI environments. Overall, the findings of the three experiments suggest that visual-spatial processing is different in immersive and non-immersive environments, and that immersive environments may require different image encoding and transformation strategies than the two other non-immersive environments. Specifically, in a non-immersive environment, participants may utilize a scene-based frame of reference and allocentric encoding whereas immersive environments may encourage the use of a viewer-centered frame of reference and egocentric encoding. These findings also suggest that MR performed in laboratory conditions using a traditional 2D computer screen may not reflect spatial processing as it would occur in the real world.

## Introduction

Our ability to generate and transform three-dimensional (3D) visual-spatial images is important not only for our every-day activities (locomotion, navigation) but also for a variety of professional activities, such as architecture, air traffic control, and telerobotics. Difficulties of studying visual-spatial cognition within real world environments, where controlling the experimental stimuli and recording participants’ behavior is often impossible, have led researchers to increasingly employ 3D immersive (3DI) virtual environments (Chance et al., 1998; Klatzky et al., 1998; Loomis et al., 1999; Tarr and Warren, 2002; Macuga et al., 2007; Kozhevnikov and Garcia, 2011). Specifically, 3DI technology allows one to create a complex immersive environment of high ecological validity, in which participants are presented with and manipulate a variety of 3D stimuli under controlled conditions.

An immersive virtual environment involves computer simulation of a 3D space and a human computer-interaction within that space (Cockayne and Darken, 2004). There are two major characteristics of 3DI environments that distinguish them from non-immersive 2D non-immersive (2DNI) and 3D non-immersive (3DNI) environments. First, 3DI involves *egocentric* navigation (the user is surrounded by the environment) rather than *exocentric* navigation where the user is outside the environment, looking in. Second, unlike non-immersive environments where a scene is fixed on a 2D computer screen, 3DI involves image updating achieved by position and head orientation tracking. Although little is known about cognitive processes and neural dynamics underlying image encoding and transformation in 3DI environments, researchers have speculated that immersivity would differentially affect selection of a spatial frame of reference (i.e., spatial coordinate system) during object encoding processes (Kozhevnikov and Garcia, 2011).

Two different spatial frames of reference, *environmental* and *viewer-centered*, can be used for encoding and transforming visual-spatial images. An environmental frame may involve the “permanent environment” which is bound by standard orthogonal planes, i.e., the floor, walls, ceiling, and perceived direction of gravity or the local “scene-based” spatial environment where the target object’s components are encoded allocentrically in relation to another object, i.e., table-top, blackboard, computer screen, etc. In contrast to environmental frames of reference, the viewer-centered frame is egocentric, that is, it defines object configurations and orientations relative to the viewer’s gaze and it includes an embedded retinal coordinate system. In the case of imagined spatial transformations such as mental rotation (MR), the prevailing hypothesis is that individuals rely more upon an environmental, scene-based, rather than a viewer-centered frame of reference (Corballis et al., 1976, 1978; Rock, 1986; Hinton and Parsons, 1988; Palmer, 1989; Pani and Dupree, 1994). For example, Corballis et al. tested normal-mirror discriminations of rotated alphanumeric characters when participants’ heads or bodies were either aligned with the gravitational vertical or misaligned by up to 60°. The results showed that the participants made their judgments by rotating the characters to the gravitational vertical (*Y* axis) rather than using a viewer-centered (head-centered or retina-centered) reference frame. Furthermore, Hinton and Parsons (1988) reported that while mentally rotating two shapes positioned on a table into congruence, participants often rotated one shape until it had the same relationship to the table-top (and room) as the other shape (thus achieving scene-based alignment), even though this produced quite different retinal images. Thus, it appears that the orientation of the viewer is defined relative to the scene, rather than the orientation of the scene being defined relative to the viewer. This lends support for theories suggesting that the representation of spatial relationships is established primarily in terms of scene-based reference systems.

**Figure 1:**
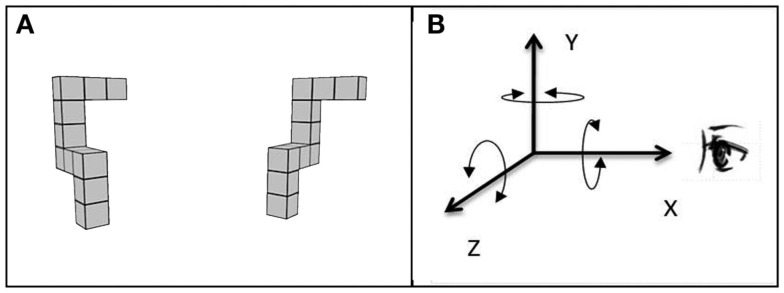
**Example of the MR test trial stimulus with (A) showing the subject’s view of the stimulus and (B) showing the three principle axes of rotation, *X*, *Y*, and *Z*, used in the current study**. Note that this coordinate frame differs from the one normally used in computer graphics in which the positive *Z*-direction is perpendicular to the plane of the display, pointing toward the viewer.

Additional evidence for primacy of scene-based reference frames comes from experiments (e.g., Parsons, 1987, 1995) comparing the speed of MR of classical Shepard and Metzler’s (1971) 3D forms around different axes (see Figure 1A). MR around different axes places different demands on the transformation processes, and results in different brain activity (Gauthier et al., 2002). Rotation in the picture plane preserves the feasibility of all the features of a shape, but perturbs the top-bottom relations between features. Rotation in depth around the vertical axis alters side-to-side relationships between features and the visibility of features, some coming into view and others becoming occluded. Rotation in depth around a horizontal axis is the most demanding rotation; it alters top-bottom relations between features and feature visibility. Interestingly, it has been consistently found that participants mentally rotate shapes in the depth plane just as fast as or even faster than in the picture plane (Shepard and Metzler, 1971; Parsons, 1987, 1995). If participants were in fact rotating viewer-centered 2D retina-based visual representations, the depth rotation would take longer than rotation in the picture plane since rotation in depth would have to carry out additional foreshortening and hidden line removal operations, not required during picture plane rotation.

Shepard and Metzler (1971) were the first to interpret similar slopes for rotation in depth and in the picture plane to indicate that latency was a function of the angle of rotation in three dimensions, not two, as in a retinal projection (for additional discussion see Pinker, 1988). In order to investigate this further, Parsons (1987) conducted an extensive experimental study examining the rates of imagined rotation not only around three principal axes of the observer’s reference frame, but also around diagonal axes lying within one of the principal planes (frontal, midsagittal, or horizontal) and around “skew” axes not lying in any of the principal planes. The findings indicated that the rotation around different axes, including rotation in depth around a horizontal axis perpendicular to the line of sight (*Z* axis, see Figure 1B) were as fast as or even faster than rotations in the picture plane (rotations around the axis defined by the line of sight, *X*-axis in this study). Parsons concluded that this equal ease of rotating images around different axes support scene-based encoding, during which the observers rely largely on representations containing more “structural” information (e.g., information about spatial relations among the elements of the object and their orientations with respect to the scene in which the objects lie) rather than on retina-based 2D representations of visual-spatial images.

One limitation of previous studies on MR is that they have been conducted using traditional non-immersive environments, where the stimuli were presented on a 2D computer screen or another flat surface (e.g., a table-top), which defines a fixed local frame of reference. This limited and fixed field of view (FOV) may encourage the use of a more structural scene-based encoding, during which the parts of the 3D image are encoded in relation to the sides of the computer screen or another salient object in the environment. However, because 3DI environments enclose an individual within the scene and allow images to be updated with respect to the observer’s head orientation, egocentric, viewer-centered encoding may predominate.

The primary goal of the current research was to examine how individuals process visual-spatial information (specifically encode and rotate 3D images) and what spatial frames of reference they rely upon in 3DI virtual environments vs. conventional non-immersive displays. In our first experiment, in order to control the effect of “three-dimensionality” vs. “immersivity,” we compared participants’ performance on the Shepard and Metzler (1971) MR task across the following three types of environments; traditional 2DNI, 3DNI (anaglyphic glasses), and 3DI [head mounted display (HMD) with position and head orientation tracking]. In the second experiment, we compared how participants encode and transform visual-spatial images in different 3DI environments with different backgrounds where shapes were embedded in a realistic scene vs. in a rectangular frame. Furthermore, if the neurocognitive correlates of visual-spatial imagery are affected by immersivity of visual presentation environment, this should be evidenced in the underlying temporal dynamic and/or spatial distribution of the electroencephalogram (EEG) response. Thus, in the third experiment, EEG was recorded while participants performed the MR task in 3DI and 2DNI environments.

## Experiment 1

### Materials and methods

#### Participants

Fourteen volunteers (eight males and eight females, average age = 21.5) participated in the study for monetary compensation. The study was approved by George Mason University (Fairfax, VA, USA) as well as by The Partners Human Research Committee (PHRC, MA, USA) and informed consent was obtained from all subjects. Participants were asked about their ability to perceive stereoscopic images prior to the start of the experiment, and only those who did report difficulty with stereopsis were included.

#### Materials and design

Each participant completed the MR task – a computerized adaptation of Shepard and Metzler’s (1971) task – in three different viewing environments: 3DI, 3DNI, and 2DNI. For each trial, participants viewed two spatial figures, one of which was rotated relative to the position of the other (Figure 1A). Participants were to imagine rotating one figure to determine whether or not it matched the other figure and to indicate whether they thought the figures were the same or different by pressing a left (same) or right (different) button on a remote control device. Participants were asked to respond as quickly and as accurately as possible. Twelve rotation angles were used: 20, 30, 40, 60, 80, 90, 100, 120, 140, 150, 160, and 180°. The figures were rotated around three spatial axes: line of sight (*X*), vertical (*Y*), and horizontal (*Z*) corresponding to rotations parallel with the frontal (*YZ*), horizontal (*XZ*), and midsagittal (*XY*) anatomical planes, respectively (Figure 1B). The test included: 12 trial groups for the 12 rotation angles, 3 trial pairs for the 3 axes, and each pair had 1 trial with matching figures and 1 trial with different figures; thus, there were 72 (12 × 3 × 2) trials in total.

**Figure 2:**
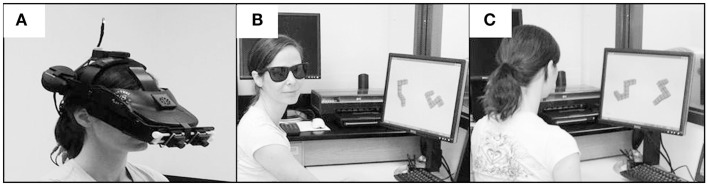
**Three different viewing environments (A) 3DI, which includes HMD with position tracking, (B) 3DNI with anaglyphic glasses to present a stereo picture of three-dimensional spatial forms, and (C) 2D monocular viewing environment**.

In the 3DI virtual environment, the shapes were presented to the participant through an nVisor SX60 (by Nvis, Inc.) HMD (Figure 2A). The HMD has a 44° horizontal by 34° vertical FOV with a display resolution of 1280 × 1024 and under 15% geometric distortion. During the experiment, participants sat on a chair in the center of the room, wearing the HMD to view “virtual” Shepard and Metzler images in front of them. Sensors on the HMD enabled real-time simulation in which any movement of the subject’s head immediately caused a corresponding change to the image rendered in the HMD. The participant’s head position was tracked by four cameras located in each corner of the experimental room and sensible to an infrared light mounted on the top of the HMD. The rotation of user’s head was captured by a digital compass mounted on the back of the HMD.

In the 3DNI environment, the shapes were presented to the participant on a computer screen. Stereoscopic depth was provided by means of anaglyphic glasses (Figure 2B). In the 2DNI environment, the shapes were presented for on a standard computer screen (Figure 2C).

The retinal image size of the stimuli was kept constant across all the environments (computed as ratio of image size over the participant’s distance to the screen). The Vizard Virtual Reality Toolkit v. 3.0 (WorldViz, 2007) was used to create the scenes and to record the dependent variables (latency and accuracy).

Before beginning the MR trials, participants listened to verbal instructions while viewing example trials in each environment. Eight practice trials were given to ensure participants’ comprehension of the instructions and that they were using a MR strategy (as opposed to a verbal or analytical strategy). If a response to a practice trial was incorrect, the participants were asked to explain how they solved the task in order to ensure the use of a rotation strategy (i.e., rather than verbal or analytical strategy). In 3DI, to familiarize the participants with immersive virtual reality, there was also an exploratory phase prior to the practice trials in which the participants were given general instructions about virtual reality and the use of the remote control device (about 7–10 min). During the practice and test phases, the participants remained seated in the chair, but were allowed to move and rotate their head to view 3D Shepard and Metzler shapes. The participants were also given similar time to familiarize themselves with the shapes in the 3DNI and 2DNI environment, and were also allowed to move and rotate their head to view Shepard and Metzler shapes.

### Results

Descriptive statistics for performance in the three environments are given in Table 1. Outlier response times (RTs; i.e., RTs ± 2.5 SD from a participant’s mean) were deleted (a total of 2.59% of all trials). All simple main effects were examined using the Bonferroni correction procedure. Two participants that performed below chance level were not included in the analysis, thus the final analysis was performed on 12 participants only.

**Table 1:** **Descriptive statistics for three versions of the MR test in 2DNI, 3DNI, and 3DI**.

Test	Proportion correct	SD	RT (s)	SD
2D	0.90	0.07	5.33	1.02
3D non-immersive	0.86	0.10	5.47	1.64
3D immersive	0.87	0.09	5.42	1.46

Response accuracy (proportion correct) and RT for correct responses were assessed as a function of the rotation axis (*X*, *Y*, and *Z*) and environment (3DI, 3DNI, and 2DNI). Data were analyzed using a 3 (axis) × 3 (environment) repeated measures ANOVA with a General Linear Model (GLM). The effect of environment was marginally significant [*F*(2,22) = 2.9, *p* = 0.040] and as pairwise comparison showed, the accuracy in 3DNI and 3DI environments was slightly less than in 2DNI (*p* = 0.08). There was a significant main effect of axis [*F*(2,22) = 19.83, *p* < 0.001] where *Y* axis rotations were more accurate than *X* and *Z* axis rotations (*p*s < 0.01). The interaction was not significant (*F* < 1). Overall, the accuracy level was relatively high for all the environments and all axes, with the proportion correct ranging from 0.84 to 0.97. Given the high rate of accuracy, indicating that ceiling performance was reached for some rotations, we focused our remaining analyses on the RTs.

**Figure 3:**
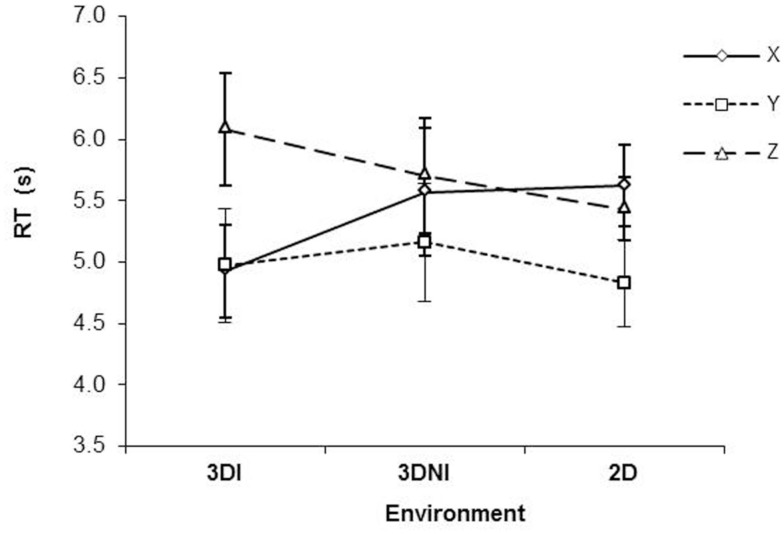
**Response time as a function of axis of rotation and viewing environment (2DNI, 3DNI, and 3DI)**. Error bars represent standard error means.

With respect to RT, there was a significant effect of axis [*F*(2,22) = 15.40, *p* < 0.001] with *Y* axis rotations being the fastest (*p*s < 0.05), see Figure 3. There was no significant effect of environment (*F *< 1), however, there was a significant interaction between axis and test environment [*F*(4,44) = 6.45, *p* < 0.001]. Analysis of simple main effects revealed that, RT for rotation around the *Y* axis was significantly faster than either around *X* (all *p*s < 0.05) or *Z* (all *p*s < 0.05) for 3DNI and 2DNI environments, consistently with previous studies (Shepard and Metzler, 1971; Parsons, 1987, 1995). However, rotations around *X* and *Z* axes were similar (*p* = 0.98 and 0.79 for 2DNI and 3DNI respectively). Interestingly the opposite occurred for MR in the 3DI environment. In 3DI, rotation around *Z* was significantly longer than *X* (*p* = 0.001) or *Y* (*p* = 0.01), while rotations around *X* and *Y* were similar (*p* = 0.97).

Thus, our central finding is that in 3DI, the RT of rotation differed between *X* and *Z* axes (*Z* was slower) and that rotation around the *Y* axis was faster than *Z* but not faster than *X* rotations. In contrast, RT patterns for 2DNI and 3DNI environments were similar to those found in previous MR studies (i.e., *Y* rotations are faster than *X* and *Z* and *X* and *Z* are similar).

#### 

##### Rate of rotation as a function of axis and environment

**Figure 4:**
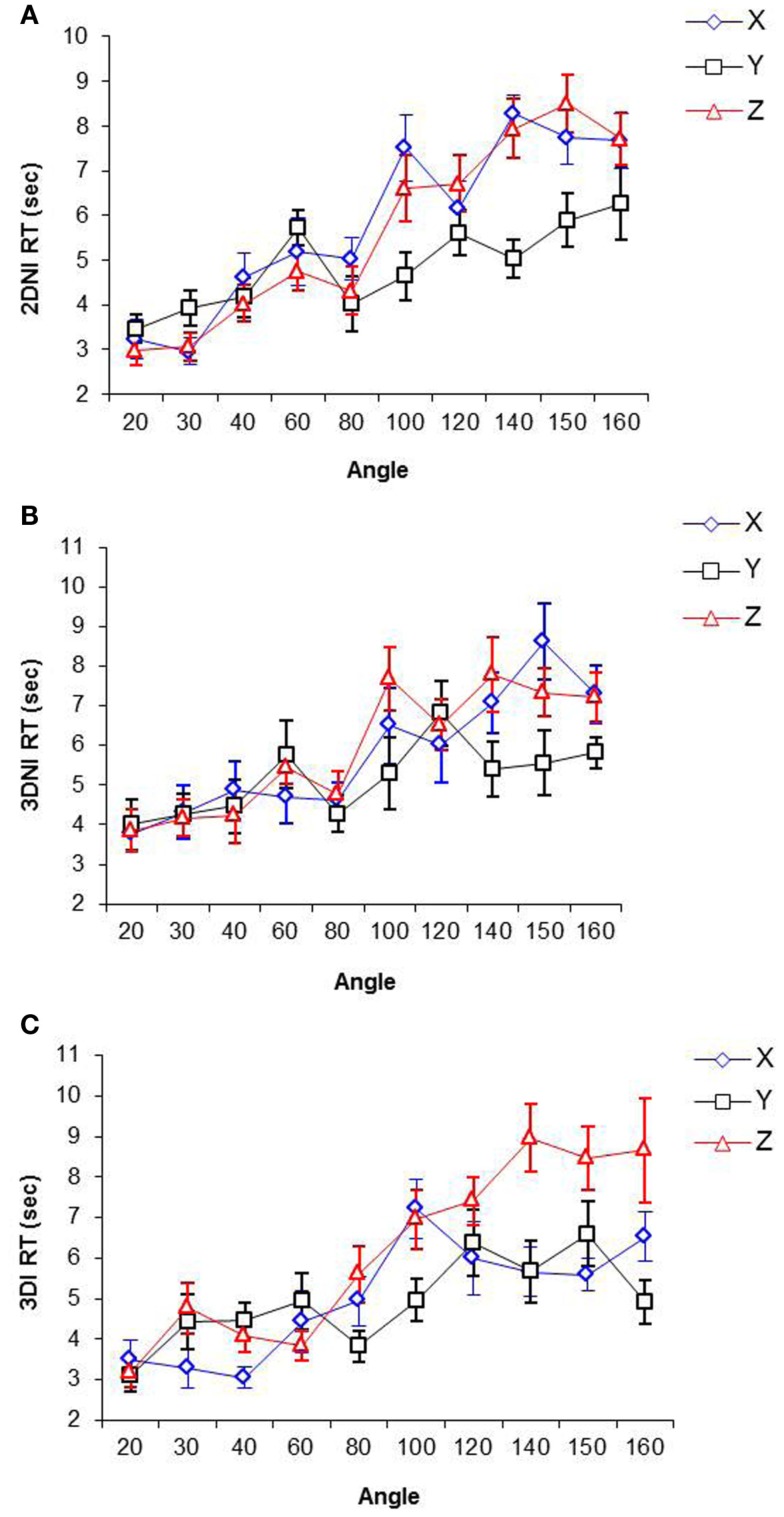
**Response time as a function of angle and axis of rotation in (A) 2DNI, (B) 3DNI, and (C) 3DI environments**.

RT as a function of rotation angles (i.e., orientation differences between two Shepard and Metzler shapes) around *X*, *Y*, and *Z* axes for 3DI, 3DNI, and 2DNI environments respectively are shown in Figure 4. The range of rotation angles was from 20 to 160; 180° was omitted due to participant’s reports that for this particular angle, they did not rotate shapes mentally, but only scanned two images for mirror-reversed symmetry.

The slopes of the best-fit linear RT-Rotation Angle functions for each axis and each environment (representing *rates of rotation* around different axes in different environments) were computed and are presented in Table 2.

**Table 2:** **Mean regression slopes of RT-Rotation angle function (s/°)**.

Environment	Axis of rotation		
	*X*	*Y*	*Z*
3DI	0.028	0.014	0.043
3DNI	0.029	0.013	0.031
2DNI	0.032	0.016	0.036

A repeated measures ANOVA of slopes of best-fit linear regression equations of RT on Rotation Angle show a significant effect of axis on the slope [*F*(2,22) = 51.34, *p* < 0.001] and a significant interaction between environment and axis of rotation [*F*(4,44) = 3.38, *p* < 0.05], while the effect of environment is not significant. For 3DI, the rate of rotation around *Z* was more than 1.5 times slower than around *X* (*p* < 0.05). In both 3DNI and 2DNI, the rate of rotation around *X* and *Z* did not differ. Across the environments, the rate of rotation around *X* was significantly faster for 3DI than for 2DNI (*p* < 0.05), whereas the rate of rotation around *Z* was significantly slower for 3DI than for either 3DNI or 2DNI (*p*s < 0.05). There were no significant differences in the rate of rotation around the *Y* axis across environment, and the rate of rotation around *Y* seems to be the one of the fastest rotations. This is consistent with the findings of previous investigators (Rock and Leaman, 1963; Attneave and Olson, 1967; Parsons, 1987; Corballis, 1988) who argued that rotation around *Y*, a “gravitational vertical” axis, is the most common of all rotations in our ecology, so that the fast rate of rotation around it may result from our extraexperimental familiarity.

In summary, the results of Experiment 1 show that the rate of MR about the horizontal axis (*Z* axis) in 3DI (and only 3DI) was significantly slower than the rate of rotation about the line of sight (*X*-axis). This finding suggests that in the 3DI environment the participants were encoding and rotating 2D retina-based visual representations in relation to a *viewer-centered* frame of reference since only then would depth rotation take longer than rotation in the picture plane, due to the involvement of additional foreshortening and hidden line removal transformations. In contrast, in 2DNI and 3DNI environments, the rates of MR around the *X* and *Z* axes were not different, consistent with previous findings for MR using 2D traditional computer displays (Shepard and Metzler, 1971; Parsons, 1987). Thus, in non-immersive environments, participants seem to generate visual representations containing more allocentric information such as information about spatial relations among the elements of the object and their orientations with respect to the scene (i.e., the computer screen) in which the object is presented. The fact that there was equivalent performance in 2D and 3DNI environments suggests that depth information *per se*, which is provided in a 3DNI environment is insufficient to encourage the use of viewer-centered frame of reference.

## Experiment 2

One of possible limitations of Experiment 1 is that, in the 3DI environment, the Shepard and Metzler shapes were presented to participants on a non-realistic “empty” background lacking any points of reference (such as ceilings, walls, other objects), which would usually be present in a real scene. Thus, viewer-centered encoding observed in 3DI could be due not to the immersivity of the environment, but rather due to the lack of any other objects – except the observers themselves – in relation to which Shepard and Metzler shapes could have been encoded. In Experiment 2, the participants in a 3DI condition were presented with Shepard and Metzler forms embedded in a realistic scene (city). In addition, we added a second condition in which the participants viewed Shepard and Metzler forms embedded in a virtual rectangular-shaped frame within the 3DI environment. This was done to examine whether the fixed frame around objects in a 3DI environment induces scene-based encoding similar to a computer screen in the real world.

### Materials and methods

#### Participants

Twenty-six volunteers (10 males and 16 females, average age = 20) recruited by advertisement participated in the study for monetary compensation. The study was approved by George Mason University (Fairfax, VA, USA) as well as by The Partners Human Research Committee (PHRC, MA, USA) and the informed consent was obtained from all subjects. Participants who reported difficulty with stereopsis were excluded from participation.

#### Materials and design

**Figure 5:**
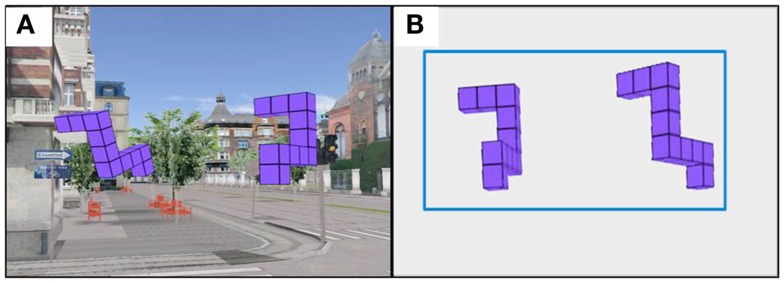
**Two different viewing environments (A) 3DIC (3DI City) environment, (B) 3DF (3D Frame) environment**.

Each participant completed the MR task used in Experiment 1 in three different viewing environments: 3DIC (3DI City) environment, where Shepard and Metzler forms were embedded in a realistic scene of a city (Figure 5A), 3DF (3D Frame), where Shepard and Metzler forms where embedded in a rectangular frame (Figure 5B), and a 2DNI environment. The order of environments was counterbalanced. The experimental procedure was similar to that described in Experiment 1.

### Results

Descriptive statistics for proportion correct and RT correct for 2DNI, 3DF, and 3DIC environments are given in Table 3. Outlier RTs (i.e., RTs ± 2.5 SD from a participant’s mean) were deleted (a total of 3.84% of all trials). One participant that performed below chance level was not included in the analysis.

**Table 3:** **Descriptive statistics for the MR test in 2DNI, 3DF, and 3DIC (*N* = 25)**.

Environment	Proportion correct	SD	RT correct (s)	SD
2DNI	0.81	0.09	5.88	1.76
3DF	0.76	0.09	6.19	1.87
3DIC	0.75	0.08	6.32	1.76

The overall accuracy (proportion correct) ranged from 0.75 to 0.81. For accuracy, a 3 (axis) × 3 (environment) repeated measures ANOVA revealed a significant effect of axis [*F*(2,48) = 5.38, *p *< 0.01], where *Z* axis rotations were significantly less accurate than either *Y* or *X* (*p*s < 0.05). There was a significant effect of environment *F*(2,48) = 13.59, *p *< 0.001]. Participants were significantly less accurate in the 3DIC environment than in either the 2DNI or 3DIF environments (*p*s < 0.01), while 2DNI and 3DIF did not differ among themselves. Also, there was a significant interaction between axis and environment [*F*(4,96) = 26.04, *p *< 0.001]. Accuracy for rotation around the *Y* axis was higher than for rotations around *X* in 2DNI (*p *< 0.01), but there was no differences between the accuracy of *X* and *Y* rotation in 3DF and 3DIC.

**Figure 6:**
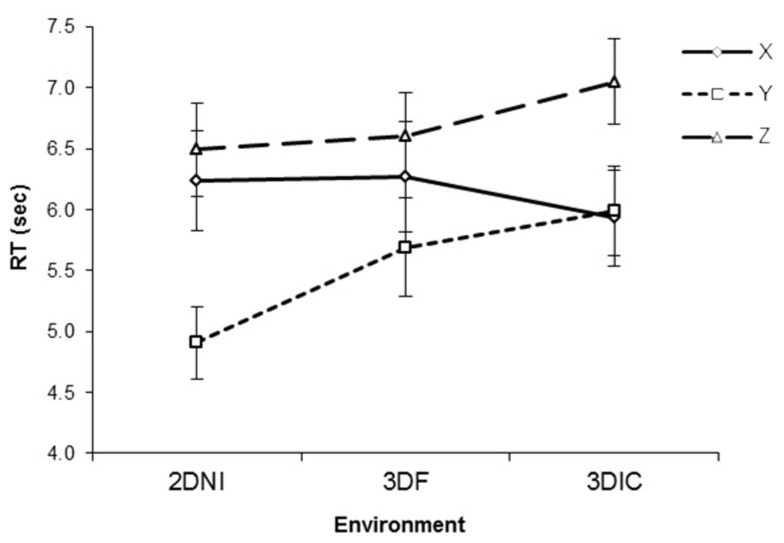
**Response time as a function of axis of rotation and viewing environment (2DNI, 3DIF, and 3DIC)**. Error bars represent standard error means.

Figure 6 presents RT against viewing environment for each axis. For RT, there was a significant effect of axis [*F*(2,48) = 35.10, *p *< 0.001], where *Y* axis rotations were significantly faster than *Z* (*p* < 0.01) or *X* (*p* = 0.05). There was no significant effect of environment [*F*(2,48) = 3.85, *p *= 0.18]. However, there was a significant interaction between axis and test environment [*F*(4,96) = 6.18, *p *< 0.001]. Examination of simple main effects revealed that rotation around the *Y* axis was faster than around *X* in 2DNI (*p *< 0.001) and in 3DIF (*p* = 0.07). In addition, rotation around *Y* was faster than around *Z* in both 2DNI and 3DF (all *p*s* *< 0.001). However, rotations around *X* and *Z* axes were similar (*p* = 0.14 and 0.24 for 2DNI and 3DF respectively).

In contrast, for 3DIC, rotation around *Z* was significantly longer than around *X* or *Y* (*p*s* *< 0.001), while rotations around *X* and *Y* were similar (*p* = 0.78). Thus, our central finding is that in 3DIC the RT of rotation differed between *X* and *Z* axes (*Z* was slower) and that rotation around the *Y* axis was faster than *Z* but not faster than around *X*. This pattern is similar to what we reported for the 3DI environment in Experiment 1. In contrast, RT patterns for 2DNI and 3DF are similar to those found reported for 2DNI environment in Experiment 1.

In conclusion, the findings of Experiment 2 suggest that individuals encode and rotate visual-spatial representations in relation to a viewer-centered frame only in 3DI environments in which the viewer is enclosed in the scene. Presumably, the participants used a viewer-centered reference frame because they perceived themselves to be “inside” the city scene and a scene-based reference frame when they perceived themselves to be “outside” the square-shaped frame that enclosed the stimuli. The square-shaped frame is similar to the sides of the computer screen in non-immersive environments, suggesting that whenever a person is observing a scene from the “outside” (e.g., a scene is defined by the frame or computer screen), it encourages the use of “scene-based” encoding.

## Experiment 3

Previous EEG research suggests that MR task involves four sequential cognitive stages which may also differentially modulate frontal and posterior brain (Desrocher et al., 1995). The first stage at ∼200–300 ms post-stimulus is independent of the object’s angular disparity and involves early sensory processing and simple stimulus evaluation. Subsequently, at ∼300–400 ms a pre-rotation “set-up” stage involves evaluation of object orientation and rotation strategy selection. Third, is the act of MR at ∼400–800 ms post-stimulus which is followed by response selection and execution from ∼1000 ms onward.

Object encoding with respect to a specific frame of reference occurs prior to the actual process of MR. Thus, the selection of a frame of reference should begin in the earliest cognitive stages between ∼200 and 400 ms post-stimulus. Furthermore, the results of Experiments 1 and 2 demonstrate that selection of a frame of reference is determined by the viewing environment. Thus, we hypothesized that when performing a MR task in 2DNI vs. 3DI, brain response differences should be largest at early sensory and/or pre-rotation “set-up” stages occurring at ∼200–400 ms post-stimulus.

### Materials and methods

#### Participants

Eight undergraduate psychology students (four males and four females) from the National University of Singapore (age between 19 and 25 years) participated in the study for monetary reimbursement. The study was approved by National University of Singapore committee and informed consent was obtained from all subjects. Participants who reported difficulty with stereopsis were excluded from participation.

#### Design and analysis

**Figure 7:**
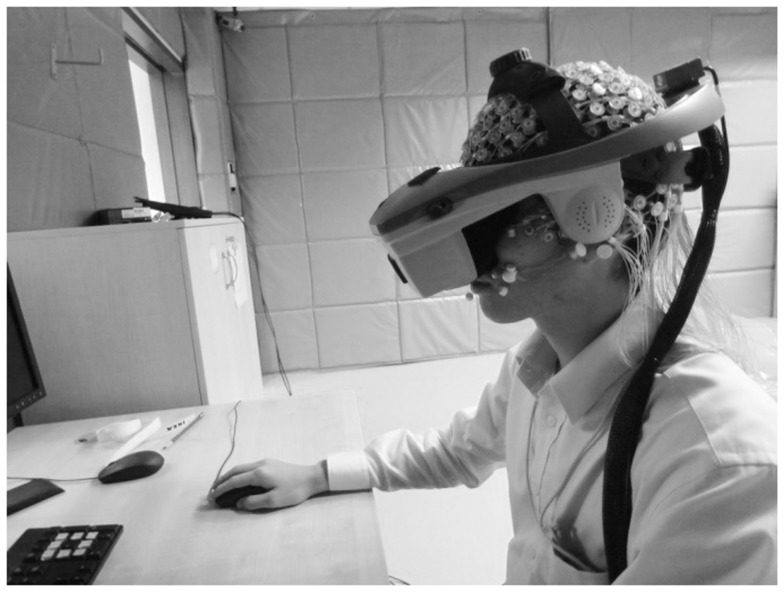
**Electroencephalogram recording in 3DI virtual reality environment**.

Electroencephalogram was recorded while subjects completed the MR task in 2DNI and 3DI viewing environments. The order of environments was counterbalanced and, in general, the procedures were similar to the first two experiments except as follows. EEG was recorded using a 256-channel HydroCel Geodesic Sensor Net (Electrical Geodesics, Inc.). Signals were amplified using the EGI NetAmps 300 amplifier. The signal was sampled at 250 Hz and bandpass filtered online at 1.0−100 Hz. For the 3DI condition, the HMD was placed directly on top of the sensor net (Figure 7).

In order to assure an adequate number of trials for averaging, participants were administered 2 blocks of 72 randomly ordered trials in the 3DI condition (overall 144 trials), and then another 2 blocks of 72 trials in the 2DNI condition.

Data preprocessing and analysis were performed using a combination of EEGLAB (Delorme and Makeig, 2004), Neuromag software (Elekta, Stockholm) and MNE Software (http://www.martinos.org/mne/). Raw EEG signals were first low-pass filtered at 40 Hz to eliminate 50 Hz electrical noise generated by the 3DI headset. Eye blink artifacts were then removed using the Signal Space Projection method provided within the open source MNE toolbox. The resultant cleaned raw data was used to create ERP averages from −200 ms pre-stimulus to 800 ms post-stimulus. Baseline was defined as −150 to 0 ms.

### Results

**Figure 8:**
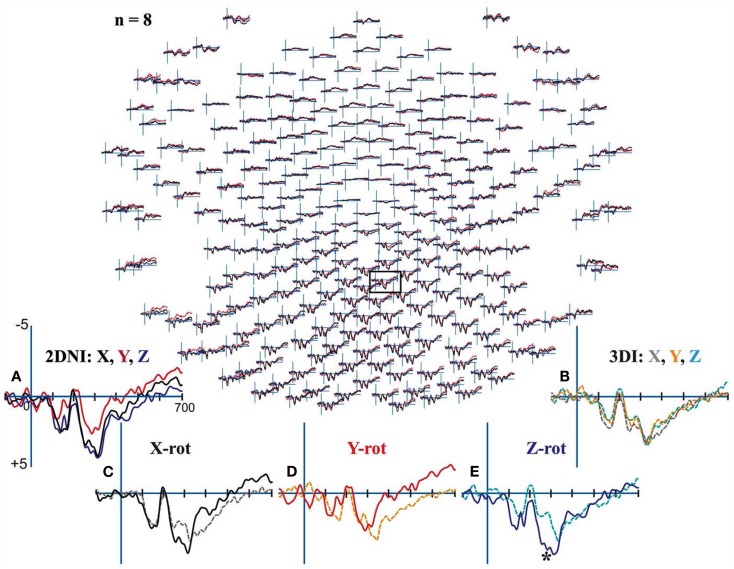
**Electroencephalograms for Mental Rotation under 2DNI and 3DI conditions: Evoked potential (μV) peaks were largest in centroparietal electrodes regardless of visual environment**. **(A)** 2DNI ERPs were most negative for *Y* axis rotations from ∼250 ms onward. **(B)** 3DI ERP’s demonstrated increasing negativities for *Z* and *Y* rotation with respect to *X* rotations at ∼350 ms. **(C–E)** When comparing rotations between 2DNI and 3DI environments 3DI rotations demonstrated a trend for greater negativity prior to 350 ms but slightly larger positivity at longer latencies >350 ms. The largest differences were between *Z*-rotations which were more positive for 3DI at ∼270–300 ms, (*) with paired *t*-test *p* < 0.03.

All participants demonstrated clear centroparietal responses while performing the MR task in the 2DNI and also the 3DI environment (Figure 8). In the 2DNI environment, parietal ERPs were highly similar for shapes rotated around the *X* and *Z* axes but more negative for *Y* axis rotations from ∼250 ms onward (Figure 8A). In the 3DI environment *X*, *Z*, and *Y* rotation demonstrated increasing negativities at ∼350 ms (Figure 8B). When comparing rotations between 3DI and 2DNI environments, rotations in 3DI showed a trend for greater negativity prior to ∼250 ms but, larger positivity at latencies >350 ms (Figures 8C–E). This effect was significant for *Z*-rotations which were more negative at ∼270–300 ms post-stimulus for MR task in the 3DI environment.

The results of Experiment 3 demonstrate that 2DNI and 3DI environments do evoke differential parietal ERP response. The early latency of these ERP differences further supports our hypothesis that 3DI and 2DNI environments differ at the level of shape encoding with respect to selection of a frame of reference. Our finding of significantly larger ERP differences for 3DI vs. 2DNI environment during *Z* axis rotations is consistent with our behavioral results from Experiments 1 and 2. Specifically, the selection of frame of reference (viewer-centered vs. scene-based) primarily affects MR in horizontal depth (*Z* axis rotation).

## Discussion

The results of this study suggest that cognitive processing in a 3DI environment differs from that occurring in 2D and 3DNI environments. Furthermore, only immersive environments seem to encourage individuals to use egocentric spatial reference frames in visual encoding and transformation tasks.

In Experiment 1 relative depth information provided by the 3DNI environment was insufficient to encourage the use of a viewer-centered frame of reference. In 2DNI and 3DNI environments parity judgments were fastest when objects were rotated around the *Y* axis, while RTs for *X* and *Z* axis rotations were similar. This suggests that the objects’ components were encoded in terms of “vertical” and “horizontal” relations with regard to the sides of the computer screen. In particular, rotation in horizontal depth plane might be relatively easy because it does not alter the orientation of the “sides” of the object with respect to the “left” and “right” sides of the computer screen (see also Parsons, 1987, for a similar discussion). This also suggests that cognition in the 2DNI and 3DNI environments is scene-based and might be atypical of human interactions with large-scale, real environments.

In contrast, our results for a 3DI environment were unique and demonstrated that viewers employed an egocentric frame of reference during MR. Specifically, the rate of rotation in horizontal depth (around *Z*) was significantly slower than that in the picture plane (around *X*). At the same time, the rate of rotation in the picture plane (around *X*) was faster in immersive environments compared to non-immersive environments, which is expected for rotation in a plane where no object components are occluded. Furthermore, when comparing between environments, rotation in the picture plane (around *X*) was the fastest in 3DI while rate of rotation in horizontal depth (around *Z*) was the slowest in 3DI. This suggests that subjects were in fact rotating a depth 2D retina-based object representations in 3DI environments and experiencing difficulties with foreshortening and occlusion.

Furthermore, the results of Experiment 2 demonstrated that the simple use of 3DI technology is not sufficient to promote the use of viewer-based frames of reference. When presentation is in 3DI and target objects are embedded within a “fixed frame,” the observer relies on scene-based encoding. This is similar to the effect of viewing in 2DNI where the fixed borders are defined by the sides of a computer screen. In this case, scene-based encoding is most efficient because the observer is free to move around without need to mentally update the position of every object within the scene. In contrast, within 3DI environments (without frame embedding) as well as in the large-scale real world, the positions of objects relative to an external frame may be constantly changing thus making viewer-centered encoding more efficient.

Finally, most previous EEG/ERP experiments on mental imagery have been performed only in 2DNI environments. The results of Experiment 3 demonstrate that 2DNI and 3DI environments do evoke differential parietal ERP responses, and that ERPs were more negative at ∼270–300 ms post-stimulus for MR in the 3DI vs. 2DNI environment. One interpretation is that this early modulation of ERP activity marks viewer-centered vs. scene-based orienting in preparation for subsequent MR from 400 ms onward. However, this early modulation may in addition involve other processes such as spatial attention and simple shape evaluation. Thus, future ERP research should evaluate the contribution of these component processes which may affect early stages of MR task performance in different visual environments. Also as Experiment 3 was a preliminary study, much work remains to be done to map the neural markers of brain processing differences between 2DNI vs. 3DI environments. This may include the use of structural MRI for anatomical localization of ERPs as well as the use of more ecologically valid task designs.

Importantly, our study is the first attempt to understand immersivity from a cognitive neuroscience perspective. Currently, there is no clear understanding of what “immersivity” means in cognitive terms. Most commonly used terms and definitions are merely descriptive, such as “perceiving oneself to be enveloped by, included in, and interacting with an environment” (Witmer and Singer, 1998, p. 227), and often confounded with such terms as “immersion” and “presence” describing the “extent to which the human operator loses his or her awareness of being present at the site and instead feels present in the artificial environment” (Durlach and Mavor, 1995, p. 22). Similarly, one problem in research aimed at understanding cognitive processing differences between virtual and real environments, is that they do not clearly define “immersivity.” For example, Perani et al. (2001) reported that the right inferior parietal cortex (IPC) was activated when subjects observed real hand actions but not when observing hand movements in a 3D virtual environment or on a 2D display. Perani et al. suggested that within virtual environments, the right parietal system may not provide sufficient information for the computation of the spatial reference frame as it is used in the real world. However, what Perani et al. called an immersive environment was the condition in which participants laid in a PET scanner with their heads position fixed, and black curtains were arranged so that the participants were able to focus only on what was shown behind a single rectangular opening in front of them. We should note that this type of environment does not meet what we consider to be the essential requirement of “immersivity,” namely viewing the scene from the “inside.” We believe that an immersive 3D environment, in which a viewer is surrounded by the environment and no clear borders are present is necessary to encourage the use of an egocentric (viewer-centered) frame of reference.

Our findings have implications for future studies on spatial transformations of mental images and the design of testing environments. They show that the results of the previous experiments on MR, performed in laboratory conditions using a traditional 2D computer screen, might be limited in that they may not reflect the MR patterns that would be measured in a natural, 3D environment. In addition to its theoretical implications, this research could be of considerable interest from an applied perspective; specifically for the design of training and learning environments. Although 3D environments might be more attractive to the user, the results of the current research show that there will probably be no significant differences between encoding and spatial transformation of images under 2DNI and 3DNI conditions. On the other hand, a 3DI environment can provide a unique and possibly more realistic learning environment. In particular, a 3DI environment should provide advantage to those tasks that benefit from encoding from an egocentric frame of reference (e.g., navigation, wayfinding, laparoscopic surgery, and telerobotics). In general, using desktop graphics to train users for real world egocentric spatial tasks might not be effective, and may actually be counterproductive due to the differences in encoding and transformation processes in immersive vs. non-immersive environments. In fact, the findings of this research explain the results of previous studies that show no transfer from training in 2D non-environments to immersive virtual environments. For instance, Pausch et al. (1997) reported that immersive prior practice with conventional 2D displays in visual search tasks impaired performance in immersive virtual environments. The researchers suggested that using desktop graphics to train users for real world search tasks may not be efficient. The current study explains this finding by pointing out that the encoding of spatial relations and cognitive strategies applied to perform visual/spatial transformations in these two types of environments are different. We suggest that 3DI environments with a variety of simulated 3D stimuli will provide the most efficient environment for training egocentric visual-spatial skills that will generalize and transfer to real world tasks.

## Conflict of Interest Statement

The research was conducted in the absence of any commercial or financial relationships that could be construed as a potential conflict of interest. Furthermore, the content of this article is solely the responsibility of the authors and does not necessarily represent the official views of our funding agencies.
